# Stereomicroscope with Imaging Analysis: A Versatile Tool for Wetting, Gel Formation and Erosion Rate Determinations of Eutectic Effervescent Tablet

**DOI:** 10.3390/pharmaceutics14061280

**Published:** 2022-06-16

**Authors:** Pornsit Chaiya, Siriporn Okonogi, Thawatchai Phaechamud

**Affiliations:** 1 Department of Pharmaceutical Technology, Faculty of Pharmacy, Silpakorn University, Nakhon Pathom 73000, Thailand; pornsitch@gmail.com; 2 School of Pharmacy, Walailak University, Nakhon Si Thammarat 80160, Thailand; 3 Research Center of Pharmaceutical Nanotechnology, Faculty of Pharmacy, Chiang Mai University, Chiang Mai 50200, Thailand; siriporn.okonogi@cmu.ac.th; 4 Department of Pharmaceutical Sciences, Faculty of Pharmacy, Chiang Mai University, Chiang Mai 50200, Thailand; 5 Natural Bioactive and Material for Health Promotion and Drug Delivery System Group (NBM Group), Faculty of Pharmacy, Silpakorn University, Nakhon Pathom 73000, Thailand

**Keywords:** wetting rate, gel formation rate, erosion rate, effervescent tablet, stereomicroscope

## Abstract

Wettability, gel formation and erosion behaviors could influence the drug release pattern of solid dosage forms. Typically, these parameters are evaluated using a variety of techniques. Nonetheless, there has been no previous research on versatile tool development for evaluating several tablet characteristics with a single tool. The aim of this study was to develop the versatile tool for measuring various physical properties of eutectic effervescent tablets and also investigate the relationship between these parameters with parameters from drug dissolution. Ibuprofen (IBU)-poloxamer 407 (P407) eutectic effervescent tablets were fabricated with a direct compression method. Their wetting properties, gel formation and erosion behaviors were investigated using a stereomicroscope with imaging analysis in terms of the liquid penetration distance, gel thickness and erosion boundary diameter, respectively. In addition, the dissolution rate (k) and disintegration time of eutectic effervescent tablets in 0.1 N HCl buffer pH 1.2 were also determined. Incorporation of P407 into the IBU tablet improved the tablet wetting properties with increasing liquid penetration distance under stereoscope. CO_2_ liberation from effervescent agents promoted tablet surface roughness from matrix erosion. The relationship between observed physical properties and disintegration and dissolution parameters suggested that the combination of erosion by effervescent agents and gel formation by P407 had a potential influence on dissolution enhancement of the formulation. Therefore, a developed stereomicroscope with an imaging analysis technique was exhibited as an alternative versatile tool for determining the wetting properties, gel formation and erosion behaviors of pharmaceutical solid dosage forms.

## 1. Introduction

Solid dosage forms are the most commonly used medication means with several advantages, and they are simple to administer with superior physicochemical stability [1,2]. Effervescent tablets are common solid dosage forms comprising effervescent agents (mixture of alkali metal bicarbonate and acid) that react upon contact with water and produce gas bubbles (carbon dioxide; CO_2_), allowing for a rapid release of the active pharmaceutical ingredient [3,4]. The rate of effervescence is impacted by a variety of parameters, including type of diluent, characteristics of binders, amount of acidic or alkaline agents used as effervescent agents and wetting properties of excipients in formulation [5,6].

The physicochemical properties of both the API and excipients used in pharmaceutical solid dosage forms must be carefully considered in order to ensure the stability and optimal performance of the formulations. For example, wettability significantly influences the various important properties such as solubility, dissolution rate and bioavailability of the drug [7]. Furthermore, the physicochemical properties have a substantial impact on its pharmacokinetic properties [8]. Wetting is a physical phenomenon that involves the displacement of a fluid (liquid or gas) from the surface of a solid or immiscible liquid by another liquid during its spreading [9]. Wetting of tablets with the gastrointestinal fluid can affect the rate of their disintegration, dispersion and dissolution. As a result, it is crucial to consider wettability as one of the key properties in designing formulations and controlling drug delivery [10]. There are various techniques, including contact angle measurement [11], the flotation method [12], isothermal microcalorimetry [13] and inverse gas chromatography (IGC) [14] for assessing the wettability of liquid on powders or pharmaceutical solid dosage forms. Typically, the most commonly used method for determination of the wettability is the measurement of the contact angle via sessile drop. However, this method has problems associated with the anomalous drop shape and penetration pattern on the surface change upon compression [15].

Empirically, wetting properties could be described from a liquid penetration perspective. A higher wetting property of formulation indicates higher liquid penetration of a medium into a tablet. Although liquid penetration does not directly enhance a tablet pressure, it is required to induce the tablet swelling or the gel layer formation [16]. Swelling and erosion characters of pharmaceutical solid dosage forms should be considered as the crucial parameters as well as the wetting properties in formulation design. Furthermore, they are reported to have considerable effects on the in vitro drug release profile of pharmaceutical dosage forms [17,18]. Typically, the type of immersion medium and polymer content influence the tablet swelling properties. High swelling due to hydration with a low erosion rate of the hydrophilic matrix tablet results in a thick gel layer. Furthermore, the strength of the gel layer impacts the diffusion coefficient of the drug; consequently, the release kinetics of the drug are significantly dependent on the swelling kinetics [19]. In general, the gravimetric (weight difference between dry and wet states of tablet) and optical observation approaches (expansion area of tablet fixing by Plexiglas) were used to investigate the swelling and erosion properties [20,21]. According to the drawbacks of conventional approaches, various advanced techniques for evaluating the swelling and erosion behaviors of the pharmaceutical solid dosage form have been developed. For example, the X-ray technique was utilized for tracking the movement of tracer molecules embedded in the gel-forming tablet. Tracer movement closing to the surface can reveal the swelling and gel development of formulations [22]. Another example is the magnetic resonance imaging (MRI) technique, which has long been recognized as a medical imaging method used in radiology to examine precise interior structures within the body. Owing to its ability to detect the ^1^H atom in water molecules, it has been used to monitor the gradients of water molecules in hydrophilic polymers and controlled drug release systems [23]. The advantages of these sophisticated approaches are typically real-time and a non-invasive method in order to minimize interfering issues. On the other hand, these approaches are commonly focused on swelling monitoring more than erosion behavior of tablets, and the expenditure on research using these advanced techniques is rather expensive [19].

To overcome the drawbacks of traditional and advanced characterization methods as previously described, the image analysis (IA) should be considered as the alternative method for investigating the wetting properties, swelling and erosion behaviors of solid dosage forms. IA is widely employed in the pharmaceutical sciences for a wide range of applications. Based on the image acquisition method used, IA can be classified as static or dynamic. Static IA is performed when the particles are stationary such as in the optical or scanning electron microscope (SEM). It should be emphasized that, for this type of IA, the restricted number of measurements, as well as the sampling size or segregation, can lead to many mistakes in the results. In contrast, dynamic IA is conducted on samples in which the particles are dispersed in a gas or liquid media, and an image is captured using a high-speed matrix or line camera. Dynamic IA results are repeatable, but they stand out in robust statistics in shorter time frames [24,25]. In case of pharmaceutical solid dosage forms, especially tablets, there are a great number of studies using IA in tablet investigation. For example, Moussa et al. investigated the axial and radial swelling kinetics on cross-sectional tablet slices after water uptake measurement. They measured the swelling as a percentage of the initial size using IA and found that the greater the degree of cross-linking of the polymer resulted in the increment of the tablet diameter [26]. Chirico et al. evaluated swelling and erosion of pure hydroxypropyl methylcellulose (HPMC), and only radial water uptake was allowed. An unexpected phenomenon was recorded using IA and found a circular halo intermediate between the erosion and the swelling radius [27]. Berardi et al. studied the disintegration behavior of single tablets in distilled water under controlled conditions and took horizontal photographs to investigate the disintegration kinetics and mechanisms [28]. As described above, the different tablet characteristics require the different IA techniques such as using a video camera with Ultimage/X software (version 1.41, Graftex, Paris, France) to investigate swelling kinetics for the study by Moussa et al. [26], using a digital camera with Mathcad to evaluate the water mass fraction for the study by Chirico et al. [27] or using a digital camera with ImagJ software (version 1.49, National Institute of Health, Bethesda, MD, USA) to observe disintegration behavior of the tablet for the study by Berardi et al. [28]. Nevertheless, there has been no previous study on the versatile IA technique that can evaluate several tablet characteristics with a single tool which is the research gap in this research field.

Therefore, to fulfill the research gap as mentioned above, this study aimed to develop a versatile IA tool for investigating the wetting properties, gel formation and erosion rate of eutectic effervescent tablets under a stereomicroscope with the IA technique. Typically, the stereomicroscope is employed to observe the morphology of pharmaceutical dosage forms such as granules [29], the microneedle patch [30] or to monitor the physical change in formulation in the transdermal drug delivery system [31]. In this study, the stereomicroscope was used to capture morphological change during immersion in a medium and, quantitative data including the rate of wetting, gel formation and erosion were extrapolated from the image sequence using OLYMPUS Stream Basic 2.2 which is an image processing program. Thus, this procedure could be a simple-to-adopt method to study the various physicochemical properties of the tablet with one single tool. The eutectic effervescent tablets comprising the eutectic mixture between ibuprofen (IBU) and poloxamer 407 (P407) and different ratios of effervescent agents were employed as model formulations. The ratio of the IBU-P407 eutectic mixture was fixed as 1:1.5 which was discovered to be the optimal ratio in a previous study [32]. The effect of effervescent agents on the wetting properties, gel formation and erosion rate of eutectic effervescent tablets was investigated under the stereomicroscope, and the correlation between these parameters with disintegration and dissolution parameters of eutectic effervescent tablets was determined to clarify the role of effervescent agents in the formulation.

## 2. Materials and Methods

### 2.1. Materials

IBU (Lot No. 4000/1101/18/A-0150B) purchased from PC Drug Co., Ltd., Bangkok, Thailand was used as the model drug, whereas P407 (Lot No. WPDF563B, BASF, Ludwigshafen, Germany) was used as the co-eutectic forming agent for IBU. The diluent in this formulation was microcrystalline cellulose PH101 (MCC PH101) (Lot No. C1910113, Mingtai Chemical Co., Ltd., Taoyuan City, Taiwan). Sodium bicarbonate (Batch No. AF310196, Ajax Finchem, Seven Hills, Australia) and citric acid anhydrous (Lot No. 90900209, Maxway Co., Ltd., Bangkok, Thailand) were employed as effervescent agents, and brilliant blue FCF (Batch No. 000BC, Yuncheng Globalchemical Biotech Co., Ltd., Yuncheng, China) was employed as the colorant for tracking penetration distance, and also the gel thickness and erosion boundary under the stereomicroscope were investigated.

### 2.2. Preparation of Eutectic Effervescent Tablets

In this study, the plain IBU tablet, IBU-P407 eutectic tablet (IP) and five different eutectic effervescent tablets (5E to 30E) were fabricated with the direct compression method. Firstly, IBU and P407 in the ratio of 1:1.5 were co-grinded in mortar and pestle for 10 min to induce eutectic formation. Then, effervescent agents (sodium bicarbonate and citric acid anhydrous in the ratio of 1:1.3 by weight) and MCC PH101 were sieved through sieve no. 20 (850 µm) into a plastic bag and mixed together with the IBU-P407 eutectic mixture for 5 min. The mixtures were compressed into 1000 mg eutectic effervescent tablets at a compression force of 2 tons and dwell time of 10 sec using 12.7 mm round, flat and plain punches using a hydraulic press (Carver press, Wabash, IN, USA). The components of eutectic effervescent tablets are illustrated in Table 1.

### 2.3. Evaluation of Eutectic Effervescent Tablets

#### 2.3.1. Investigation of Wetting, Gel Formation and Erosion Properties

**Figure 1:**
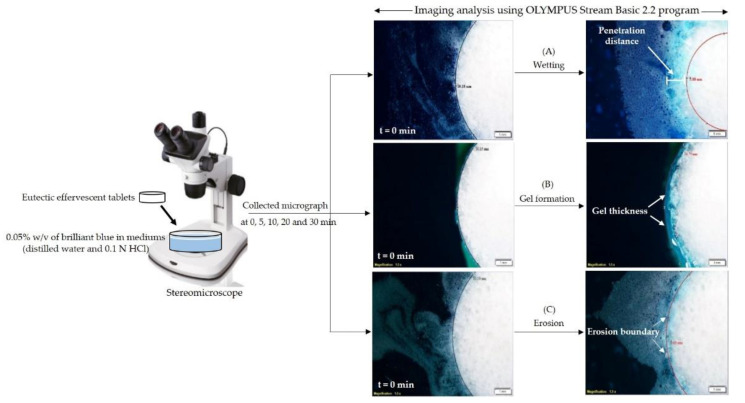
Wetting properties, gel formation and erosion investigation of eutectic effervescent tablets under stereomicroscope.

Under the stereomicroscope (Olympus model SZX10, Hamburg, Germany), the wetting properties of eutectic effervescent tablets were investigated at a magnification of 15×. Each eutectic effervescent tablet was immersed in 0.05% *w*/*v* of brilliant blue in distilled water or 0.1 N HCl buffer pH 1.2, which filled in the glass petri dish with diameter of 5.0 cm. During immersion, the morphological change in the eutectic effervescent tablet was photographed under the stereomicroscope using continuous mode at every minute for 30 min. Then, image analysis was carried out on 31 images from each sequence. The images were seen in the OLYMPUS Stream Basic 2.2 program as a single sequence of images, that is, a stack of frames, each stack covering the morphological change in the tablet at a single time point. As shown in Figure 1A, blue color penetration from the medium into the eutectic effervescent tablet was photographed. Subsequently, the penetration distance (white line) was determined from the difference between the initial border at a time point of 0 min (black line) and the wetting border at a specified time interval (red line). The slope of profile from the relationship between the penetration distance versus time was used to calculate the wetting rate of each formulation (n = 5). 

In the case of gel formation and erosion of the eutectic effervescent tablet, the experiments were carried out under the same conditions as the wetting investigation as stated above. Swelling and gel formation subsequently occurred after immersion of the prepared tablet in the medium, and gel formation of the tablet was photographed. Gel thickness was determined from the difference between the initial boundary of the tablet at 0 min (black line) and the gel boundary at a specified time interval (red line) (Figure 1B). The slope of profile from the relationship between gel thickness versus immersed time was used to calculate the gel formation rate of each formulation (n = 5). For erosion rate calculation, the erosion of each formulation was calculated from the difference between the initial border of the tablet at a time point of 0 min (black line) and the erosion boundary at a predetermined time interval (red line) (Figure 1C). Then, the erosion rate of each formulation was determined from the slope of the relationship between the erosion boundary diameter versus the immersed time profile (n = 5).

#### 2.3.2. In Vitro Disintegration Study of Eutectic Effervescent Tablets

The disintegration tester (ZT 320, Erweka, Langen, Germany) was used to measure the disintegration time (DT) of eutectic effervescent tablets, in accordance with US Pharmacopeia (USP) 43 <701> Disintegration procedures using 900 mL of distilled water at 37 ± 2 °C [33]. To investigate the effect of the pH medium on disintegration behavior of tablets and the relationship with observed parameters under the stereomicroscope, 0.1 N HCl buffer pH 1.2 was also used as the disintegration medium. Six tablets were dropped into individual tubes of the basket-rack assembly. The time at which each tablet completely disintegrated was recorded. The mean of DT ± SD was calculated (n = 6).

#### 2.3.3. In Vitro Drug Release Study of Eutectic Effervescent Tablets in 0.1 N HCl Buffer and Kinetic Parameters

According to USP43, the in vitro dissolution testing of IBU tablets should be performed using phosphate buffer pH 7.2 as the dissolution medium [34]. To determine the effect of eutectic formation on dissolution of IBU, which is a poorly water-soluble drug compound, 0.1 N HCl buffer pH 1.2 might be the discriminated medium owing to the lower solubility of IBU in acidic medium [32]. In this study, the in vitro drug release was studied in 0.1 N HCl buffer pH 1.2 using a dissolution apparatus (DT 820, Erweka, Langen, Germany) utilizing the paddle method at 37 ± 0.5 °C and a rotational speed of 50 rpm which complied with the USP43 <711> Dissolution procedure. Each 5 mL aliquot was withdrawn from the dissolution medium and replenished with 5 mL of fresh medium at a predetermined interval. An UV-vis spectrophotometer (Cary 60 UV-vis, Agilent Technology, Santa Clara, CA, USA) was used to analyze the amount of drug dissolution from tablets using the wavelength at 220 nm. The mean cumulative drug dissolution ± SD was calculated (n = 3). The mechanisms of drug release were determined by fitting the drug release data with modified Korsmeyer–Peppas and Peppas–Sahlin equations using the DDSolver software which is a menu-driven add-in program for Microsoft Excel (Redmond, WA, USA) written in visual basic applications [35]. Moreover, mathematical analysis was carried out by calculating the coefficient of determination (R^2^), Akaike Information Criterion (AIC) and Model Selection Criterion (MSC) for the drug release data with the appropriate modeling.

## 3. Results and Discussion

### 3.1. Morphological Changes in Eutectic Effervescent Tablets under Stereomicroscope

**Figure 2:**
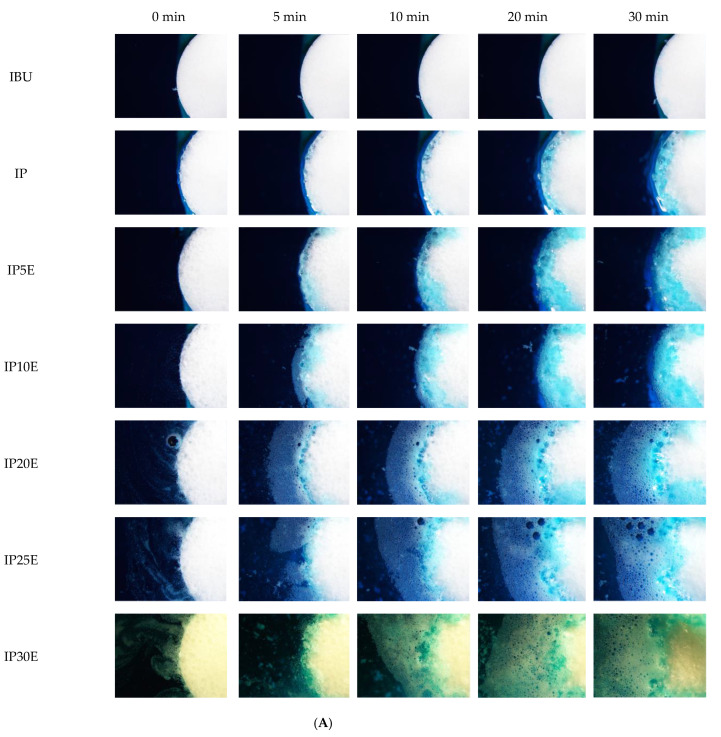

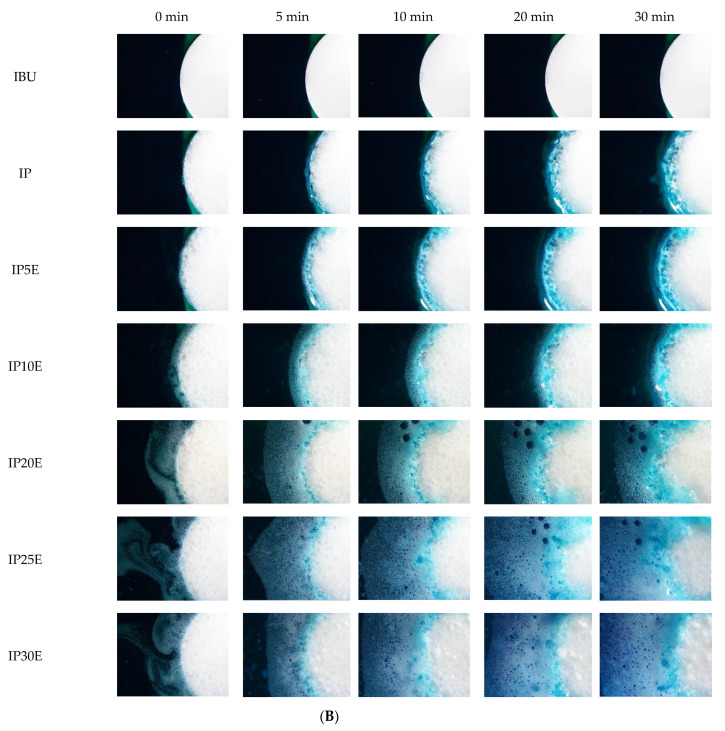
(**A**) Photomicrographs of eutectic effervescent tablets in distilled water under stereomicroscope at magnification of 15×. (**B**) Photomicrographs of eutectic effervescent tablets in 0.1 N HCl buffer pH 1.2 under stereomicroscope at magnification of 15×.

Under the stereomicroscope at a magnification of 1.5×, morphological change in eutectic effervescent tablets after immersion in distilled water and 0.1 N HCl buffer pH 1.2 at specified time intervals were photographed and are presented in Figure 2A,B, respectively. After 30 min of immersion in distilled water, there is a slight penetration of blue color into the tablet, indicating the low wetting properties of the IBU tablet in DI water (Figure 2A). Meanwhile, there has been no change in 0.1 N HCl buffer pH 1.2 (Figure 2B). This might be related to the pH-dependent solubility of IBU, as previously reported by Potthast H et al. IBU has a solubility of 3.44 mg/mL in medium pH 7.4 at 37 °C, and its solubility decreases significantly to 0.038 mg/mL in medium pH 1.0 [36]. Lower solubility in acidic medium with lower wetting properties resulted in no penetration of blue color into the IBU tablet in 0.1 N HCl buffer pH 1.2. Because of hydrophobicity and pH-dependent solubility of IBU, P407 was utilized as the eutectic co-forming agent to improve IBU solubility and wetting properties. After immersion in both testing media solutions, the penetration of blue color into a IP tablet was longer than that into a plain IBU tablet. This result supports that P407 could improve wettability and solubility of IBU which is consistent with the previous reports [32,37]. During eutectic formation, the presence of an intermolecular interaction between IBU and P407 makes it possible to lower the melting point (T_m_) and enthalpy (ΔH_f_). The decrease in ΔH_f_ indicated that solubility thermodynamically increased according to the van’t Hoff reaction. Moreover, as T_m_ decreases, the logarithmic value of the molar fraction of solute, which is IBU, approaches zero and becomes large [38,39]. Higher solubility of the IBU-P407 eutectic mixture induced the longer penetration of blue color into the IP formulation. In addition, swelling and gel formation of the IP tablet could be observed under the stereomicroscope from the gelling ability of P407 [40]. For the IP5E to IP30E formulation comprising 5–30% by weight of effervescent agents in the IP tablet, their penetration distance in both testing media revealed similar trends, with more effervescent agents promoting a greater penetration distance. When the eutectic effervescent tablets contacted the testing medium, carbon dioxide (CO_2_) gas was generated (Figure 2A,B). A higher amount of effervescent agent loading produced more CO_2_ to enhance the rough surface of eutectic effervescent tablets owing to surface erosion, allowing the medium to penetrate deeper into eutectic effervescent tablets [41]. In comparison with the traditional wetting testing method such as contact angle measurement using the goniometer, these results under the stereomicroscope clearly provided a better understanding of the wetting behavior of the eutectic effervescent tablet in both immersion media by visual observation.

### 3.2. Rate of Wetting, Gel Formation and Erosion of Eutectic Effervescent Tablets 

**Figure 3:**
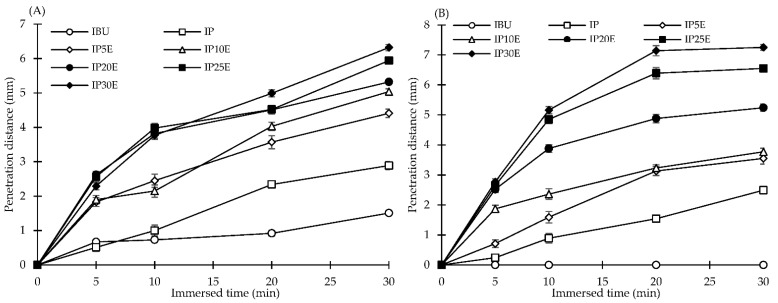
The penetration distance capacity of eutectic effervescent tablets in distilled water (**A**) and 0.1 N HCl buffer pH 1.2 (**B**).

The wetting properties were investigated using a stereomicroscope with imaging analysis by measuring the penetration distance of the blue color medium into the eutectic effervescent tablet. The wetting rate was then calculated from the slope of the relationship between the penetration distance versus time profile. The penetration distance capacity of effervescent tablets in both distilled water and 0.1 N HCl buffer pH 1.2 is shown in Figure 3. The wetting rate of each formulation was calculated from the slope of the penetration distance versus time profile as shown in Table 2. The wetting rate of eutectic effervescent tablets in distilled water increased from 0.201 to 1.459 mm/min when more effervescent agents were added from 5 to 30% by weight, respectively. Moreover, P407 could improve the wetting rate more than twice when compared with the plain IBU tablet. This can be explained by high thermodynamic functions as discussed above. When tested in 0.1 N HCl buffer pH 1.2, the wetting rate of eutectic effervescent tablets exhibited a comparable trend as distilled water but slightly lower. In the case of the plain IBU tablet, there was no penetration of blue color medium into the tablet after immersion in the medium for 30 min as illustrated in Figure 2A,B. Thus, the wetting rate of IBU was reported as the zero value in Table 2 because of high hydrophobicity and low solubility of IBU in 0.1 N HCl buffer pH 1.2 which was previously reported by Cristofoletti et al. [42]. These results were consistent with the morphological change in eutectic effervescent tablets under the stereomicroscope as previously stated. More effervescent agents in the formulation could enhance a surface roughness and allow the medium to penetrate deeper into the tablet, resulting in a higher wetting rate in formulations containing more effervescent agents. In addition, the incorporation of P407 in the IBU tablet apparently improved the wetting properties as observed with its higher wetting rate when compared with the IBU tablet.

Wetting improvement is one of the important mechanisms for enhanced dissolution of solid dispersion. It has long been recognized as a powerful technique for improving the oral bioavailability of poorly water-soluble drugs by dispersing them into inert hydrophilic polymer matrix carriers [43,44]. The sessile drop method, also known as contact angle measurement, is commonly used to estimate wettability from the interfacial interaction between solid and liquid systems. Prabhu et al. applied the co-grinding method to fabricate a solid dispersion between atorvastatin calcium and P407 and investigated its wetting properties with the sessile drop method. Pure atorvastatin calcium exhibited a high contact angle due to its hydrophobic properties. After dispersed in P407 as solid dispersion, it showed an extremely low contact angle which confirmed the significant improvement in drug solubility by P407 [45]. Łyszczarz et al. developed aripiprazole-P407 solid dispersion incorporated orodispersible films (ODFs). The contact angle value of ODFs containing aripriprazole-P407 solid dispersion were lower than placebo films which indicated an improved wettability of ODFs owing to the surface activity of P407 [46]. P407 clearly exhibited a good choice as a hydrophilic carrier in solid dispersion as well as a co-eutectic forming agent with IBU in this study. Under the stereomicroscope, higher penetration distance of the IP tablet notably signified the higher wetting property of the IP tablet as compared with a plain IBU tablet. This result exhibited a similar trend with the wetting measurement using the sessile drop method. In addition, the wetting rate was then calculated from the slope of penetration distance versus time profile and provided similar results as compared with the contact angle results in previous research studies [47,48]. Therefore, the penetration distance and wetting rate measurements from the stereomicroscope with the imaging analysis technique could be an alternative tool for the wetting investigation of pharmaceutical solid dosage forms.

**Figure 4:**
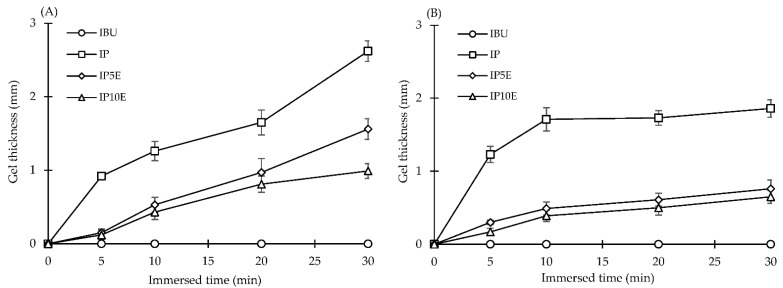
The gel formation capacity of eutectic effervescent tablets in distilled water (**A**) and 0.1 N HCl buffer pH 1.2 (**B**).

After immersion in the medium, swelling, gel formation and erosion of the eutectic effervescent tablet subsequently occurred. The stereomicroscope with imaging analysis techniques was employed to investigate the gel thickness and the erosion boundary. Gel thickness of each formulation was plotted versus immersed time, as illustrated in Figure 4. The rate of gel formation was calculated from the slope of gel thickness versus time profile and is shown in Table 2. Gel formation of eutectic effervescent tablets was determined only in three formulations including IP, IP5E and IP10E, because higher erosion rates of formulation containing more than 10% by weight of effervescent agents interfered with the determination of gel thickness under the stereomicroscope. As illustrated in Figure 4, all these three eutectic effervescent formulations showed a higher gel formation in distilled water than in 0.1 N HCl buffer, and the IP formulation showed the highest gel formation in both mediums due to the higher gelling ability of P407 in distilled water as previously reported in the report by Jannin V et al. [40]. The gel formation behavior of P407 observed under the stereomicroscope exhibited a consistent result as previously described in the wetting rate determination. Poloxamers are nonionic surfactants widely used as emulsifiers, wetting agents and solubilizers in pharmaceutical formulations. They have been incorporated into solid dispersions to improve solubility and dissolution profiles of poorly water-soluble drugs. The role of them on enhancement of drug solubility was associated with the formation of self-assembly nanoaggregates and surface activity [47]. Furthermore, adding more effervescent agents in formulations reduced the gel formation of P407 in eutectic effervescent tablets. When the effervescent agent was incorporated in the tablet, CO_2_ gas was generated and promoted an erosion of the eutectic effervescent tablet, arising in a decreased gel formation of P407 in eutectic effervescent formulation. The gel formation rate showed the same trend in both distilled water and the 0.1N HCl buffer. P407 in formulation could create a gel layer around the tablet surface, with the highest gel formation rate in distilled water being 0.184 mm/min as illustrated in Table 2. When effervescent agents were added, the gel formation rate of formulation was gradually decreased to 0.035 mm/min for the formulation containing 10% by weight of effervescent agents immersed in distilled water.

**Figure 5:**
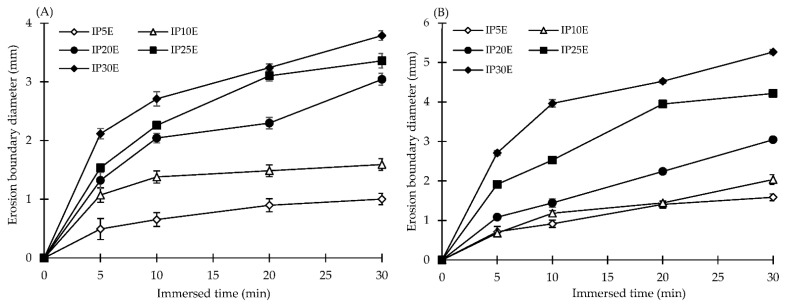
The erosion capacity of eutectic effervescent tablets in distilled water (**A**) and 0.1 N HCl buffer pH 1.2 (**B**).

The erosion capacity of eutectic effervescent tablets in both different medium solutions is shown in Figure 5. The formulation containing 5% by weight of effervescent agents exhibited an erosion distance of about 1 mm after immersion in distilled water for 30 min, and more incorporation of effervescent agents enhanced the erosion of formulations. Moreover, the erosion capacity in 0.1 N HCl buffer was evident with a similar pattern and slightly higher erosion when compared to that in distilled water. The acidic environment of the 0.1 N HCl buffer provoked more effervescence reaction and thereafter inducing more increasing erosion. Then, the erosion rate of each formulation was calculated from the slope of profile from the relationship between the erosion boundary versus immersed time as illustrated in Table 2. In terms of erosion rate, there was no erosion in IP formulation after immersion in both different medium solutions for 30 min. The erosion rate was enhanced as more effervescent agents were introduced into tablets, reaching a maximum of 0.375 mm/min when the formulation containing 30% by weight of effervescent agents was immersed in the 0.1 N HCl buffer. In a study by Desai et al., the effervescent agent has been considered as a tablet disintegrant. The volumetric air expansion from combining an organic acid with an inorganic carbonate or bicarbonate promoted disintegration of the effervescent tablets after wetting [48]. By comparison with traditional and advanced techniques for the erosion behavior investigation, the erosion boundary diameter and the erosion rate obtained from the stereomicroscope with an imaging analysis technique presented as an alternative tool in pharmaceutical product development. These techniques provide the corresponded results that the more amount of disintegrant addition promotes greater disintegration of tablets [23,49,50].

### 3.3. In Vitro Disintegration of Eutectic Effervescent Tablets

**Figure 6:**
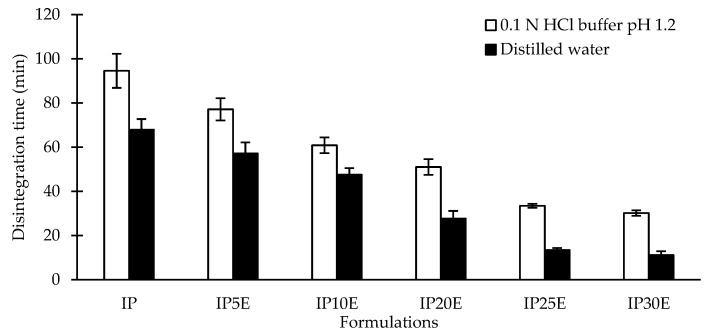
In vitro disintegration of eutectic effervescent tablets in distilled water and 0.1 N HCl buffer pH 1.2.

To investigate the effect of the pH medium on the disintegration behavior of the eutectic effervescent tablet, the in vitro DT of eutectic effervescent tablets was studied in both distilled water and 0.1 N HCl buffer pH 1.2, as illustrated in Figure 6. There is no change in the IBU tablet in both media after immersion in both media for more than 6 h (data not shown in Figure 6). With incorporation of P407 into the IBU tablet, the DT of the IP tablet dramatically decreased to 67.90 min in distilled water and 94.56 min in the 0.1 N HCl buffer. This can be explained by solubility improvement from eutectic formation and is clearly noticeable in the greater wetting rate and erosion rate as mentioned above. All formulations showed the same trends of DT in both media but slightly lesser in distilled water. As a result, solubility should be regarded as the important factor influencing the disintegration behavior of the formulation and resulting in higher dissolution of the formulation [51,52]. The carbonation reaction from effervescent agents caused the surface erosion, resulting in a deeper penetration distance of the medium and accelerated disintegration of the eutectic effervescent tablet [48,49,50]. When effervescent agents were increased from 5% to 30% by weight, the DT in distilled water dropped from 57.13 min to 11.15 min. In the case of 0.1 N HCl buffer pH 1.2, it was reduced to roughly 30 min when 30% effervescent agents were used in the IP formulation. These findings corresponded with the trend of the wetting rate and erosion rate as mentioned above.

### 3.4. In Vitro Drug Release of Eutectic Effervescent Tablet in 0.1 N HCl Buffer and Kinetic Parameters

**Figure 7:**
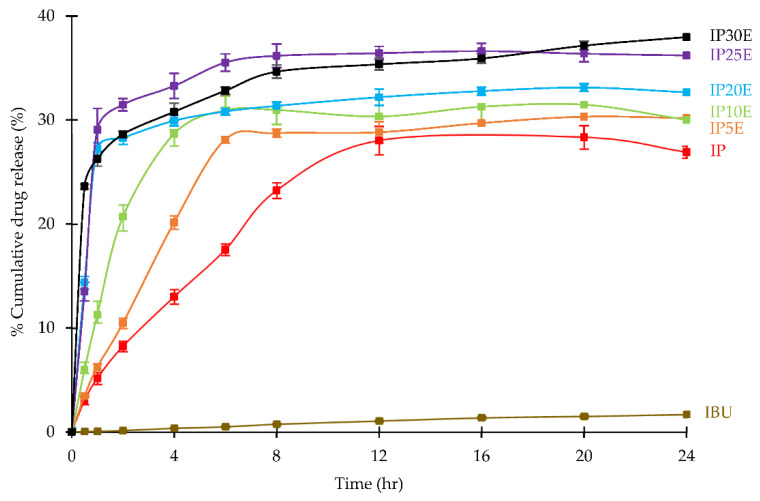
In vitro drug release profiles of eutectic effervescent tablets in 0.1 N HCl buffer pH 1.2.

The in vitro drug release profiles of eutectic effervescent tablets investigated in 0.1 N HCl buffer pH 1.2 are illustrated in Figure 7. In the case of the formulation containing effervescent agents, initial burst release (F_0_) might occur from carbonation upon contact with the dissolution medium. The modified Korsmeyer–Peppas model with F_0_ would be performed to evaluate the mechanism of IBU release from tablets, but F_0_ cannot be treated as independent variables in the Peppas–Sahlin model [35]. Thus, cumulative drug dissolution versus time profiles were fitted with both F_0_-modifed Korsmeyer–Peppas and Peppas–Sahlin models. Table 3 illustrated the mathematical modeling of IBU release from eutectic effervescent tablets. The R^2^, AIC and MSC values were used as the model selection criterion. The best model could be indicated from the highest and closest to 1.0 for the R^2^, highest value for MSC and lowest AIC [35]. From the results, F_0_ obtained from modified Korsmeyer–Peppas modeling of all formulations exhibited a zero value which indicated there is no initial burst release. This appears to be inconsistent with the dissolution profile obtained, particularly in formulations containing more effervescent agents, such as the eutectic tablet containing 30% effervescent agents, which showed about 25% dissolved drug and about 15% dissolved drug for 25% effervescent agents in the eutectic tablet within 30 min. In comparison with the study by Andhariya et al., this seems to be a slightly initial burst release of IBU from the eutectic tablet containing high amounts of effervescent agents [53]. Typically, carbonation reaction would occur immediately upon contact with the medium, resulting in faster disintegration of the formulation. As seen with the results, effervescent tablet should have a high initial burst release. In this study, as observed under the stereomicroscope, the carbonation reaction of eutectic effervescent tablets gradually occurred upon immersion in the medium. This is explained by gelling capabilities of P407, which can retard the breakdown of the eutectic effervescent tablet [54]. For the results of mathematical model fitting, all formulations exhibited the tendencies of Peppas–Sahlin in which the R^2^ values were close to 1.0. The dissolution rate can be obtained from the positive k value from the Peppas–Sahlin model [55]. The plain IBU tablet showed the lowest dissolution rate of 0.3694% dissolved drug/h, with heterogenous erosion as the predominant drug release mechanism, as indicated by the positive k_2_ value as shown in Table 3. In comparison to the plain IBU tablet, IP incorporation enhanced the dissolution rate to 5.1761% dissolved drug/h and exhibited the maximum dissolution of about 28% at 24 h. The effervescent agent added into the IP formulation increased the dissolution rate from about 8% dissolved drug/h for 5% by weight of effervescent agents to 32% for 30% by weight of effervescent agents. Moreover, 30% by weight of effervescent agents promoted the maximum dissolution by up to 38% (Figure 7). The kinetic parameters were estimated using the Peppas–Sahlin model fitting, and the release mechanism is indicated by a higher value when comparing the k_1_ and k_2_ value. The higher k_1_ value suggested that the diffusion process was the predominant drug release mechanism, whereas higher k_2_ indicated that poloxamer relaxation or heterogenous erosion was the main drug release mechanism [55]. Except for the IBU tablet, all formulations indicating a positive k_1_ value suggested that IBU release could be explained by the diffusion process. Additionally, the addition of effervescent agents to the IP tablet did not affect the IBU release mechanism. These findings supported a slightly initial burst release which was observed in eutectic effervescent tablets mentioned above. Upon contact with the medium, effervescent agents could promote the breakdown of the tablet, and small granules were suspended in the medium. Some fractions of granules were disintegrated and released as an initial burst drug release; remaining fractions could be swelling owing to gelling properties of P407 and facilitating the drug release via diffusion [56,57].

### 3.5. Correlation between Rate of Wetting, Gel Formation and Erosion with Disintegration and Dissolution Parameters 

**Figure 8:**
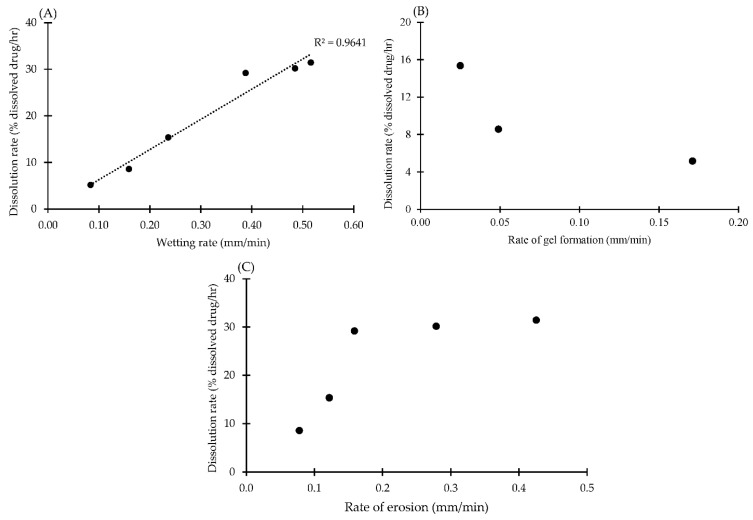
Correlation between rate of wetting (**A**), gel formation (**B**) and erosion (**C**) with dissolution rate of eutectic effervescent tablets in 0.1 N HCl buffer pH 1.2.

**Figure 9:**
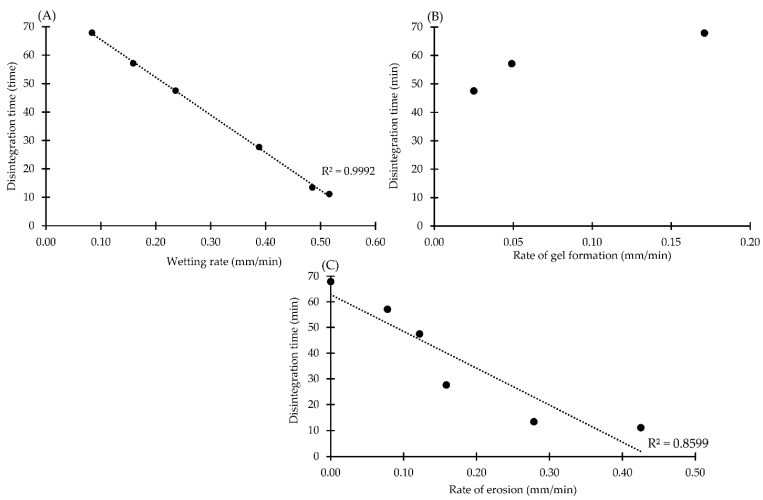
Correlation between rate of wetting (**A**), gel formation (**B**) and erosion (**C**) with disintegration time of eutectic effervescent tablets in 0.1 N HCl buffer pH 1.2.

To clarify the main mechanism of dissolution rate improvement in effervescent formulations, the wetting rate, the gel formation rate and the erosion rate were plotted with the dissolution rate and disintegration time as illustrated in Figure 8 and Figure 9, respectively. There was a positive linear relationship between the rate of wetting and dissolution rate with a high coefficient of correlation (R^2^ = 0.9641); thus, the dissolution rate of the drug was increased due to an enhancement in the wetting rate (Figure 8A). In the case of the erosion rate, the dissolution rate increased as an increment in the erosion rate in the initial period. There is no substantial change in the dissolution rate once the rate of erosion exceeds about 0.16 mm/min (Figure 8C). These findings were consistent with the investigation of the drug release mechanism using mathematical model fitting as mentioned above. After tablet breakdown, residual granules containing P407 can create a surrounding gel layer s and facilitate the drug diffusion across gel layer [58]. Typically, solubility of the soluble ingredient is affected by both wicking and disintegration of the tablet, especially in tablets containing a high concentration of soluble ingredients [50]. Wicking is the penetration of liquid by capillary action between the pore of the tablet [59] which can be compared with the wetting parameter in this study. The penetration of the liquid surrounding the tablet into the target tablet could be provoked by the eutectic mixture between IBU and P407, and this was clearly seen with a morphological change under the stereomicroscope. Higher liquid penetration promoted a higher rate of effervescence reaction in the formulation and a higher rate of erosion of IBU-P407 eutectic effervescent tablets, resulting in higher drug release as illustrated in Figure 7 [41,59]. In terms of the gel formation rate, there was no relationship between the dissolution rate and gel formation rate. This result could be explained as follows. The gel formation of P407 in the formulation was interrupted with surface erosion from generated CO_2_ gas from the effervescence reaction which was observed in the morphological change under the stereomicroscope (Figure 2B).

The correlation between the rate of wetting, gel formation and erosion with DT is presented in Figure 8. The rate of tablet wetting exhibited negative relationship with DT and could be described by a linear equation with a high R^2^ of 0.9992 as shown in Figure 8A. The 0.1 N HCl buffer was dramatically decreased from 94.56 min to 77.13 min when 5% effervescent agents were incorporated into the IP formulation and gradually declined as a decrement in the wetting rate. In terms of the rate of gel formation, DT gradually increased with an increasing gel formation rate as illustrated in Figure 8B. The erosion rate exhibited a negative relationship with DT which could be described by a linear equation as shown in Figure 8C. These findings could be explained by observing the morphological changes under the stereomicroscope. An effervescent reaction rapidly occurred when effervescent tablets came into contact with the dissolution medium. The rough surface of the tablet was evident from erosion promoting in the larger surface area of tablet, resulting in a decreasing in DT and an increasing in the drug dissolution [41]. The effervescent reaction of the effervescent tablet occurred faster than the gel formation of P407 and DT was rapidly decreased with an addition of effervescent agents. This explanation was evident as shown in Figure 8C. Nevertheless, there was no relationship between the gel formation rate and dissolution rate, but a positive relationship was found in the initial period of the graph plotted between the erosion rate and dissolution rate (Figure 8C). This could be described by the drug release mechanism obtained from mathematical modeling as discussed previously and consistent with the previous study by Zuo et al. [60]. Therefore, monitoring of tablet behaviors under the stereomicroscope with imaging analysis could explain the mechanism of dissolution rate improvement of the IBU-P407 eutectic effervescent tablet. Wetting enhancement by eutectic formation between IBU and P407 could promote the surrounding liquid penetration into eutectic effervescent tablets and promote a greater effervescence reaction. Furthermore, the stereomicroscope with imaging analysis was employed to describe the main mechanism of tablet dissolution, and it was discovered that tablet disintegration and erosion caused by carbonation of the effervescent agent and gel formation of P407 in formulations had a potential influence on drug dissolution enhancement.

## 4. Conclusions

The stereomicroscope with imaging analysis was successfully developed as a versatile tool for measuring and understanding the wetting properties, gel formation and erosion behaviors of eutectic effervescent tablets. Monitoring the penetration of the immersion medium into the tablet provided valuable information in terms of penetration distance and attainment of the gel thickness/erosion boundary diameter. These obtained values indicated gel formation and erosion behaviors, respectively. The wetting enhancement by the IBU-P407 eutectic mixture incorporated effervescent tablets denoted with greater liquid penetration distance. Effervescent agents in the tablet promoted higher erosion behavior owing to CO_2_ generation, as well as increased surface roughness. Moreover, it manifested in deeper liquid penetration into the eutectic effervescent tablet, resulting in greater disintegration and a higher rate of drug dissolution, as demonstrated in the in vitro disintegration and drug release study. The P407 gel formation surrounding tablet was declined with an increasing content of effervescent agents. Under stereomicroscope observation, an effervescence reaction rapidly occurred and disrupted the gel formation of the IP tablet. Thus, various behaviors after immersion in different media of eutectic effervescent tablets could be described. Furthermore, this technique provided the better understanding of the main dissolution mechanism of the eutectic effervescent tablet by visual observation. The correlation between several parameters attained under the stereomicroscope with disintegration and dissolution parameters suggested that a combination of erosion owing to carbonation of the effervescent agent and the P407 gel formation promotes drug dissolution enhancement. Therefore, the stereomicroscope with the imaging analysis technique exhibits as a potential versatile tool for determining the wetting properties, gel formation and erosion behaviors of solid dosage forms. Further investigation and evaluation for these behaviors with this technique should be conducted on other solid dosage forms.

## Figures and Tables

**Table 1:** Composition of eutectic effervescent tablets.

Compositions (%*w*/*w*)	Formulation						
	IBU	IP	IP5E	IP10E	IP20E	IP25E	IP30E
Ibuprofen	100.00	40.00	34.00	32.00	28.00	26.00	24.00
Poloxamer 407 (P407)	-	60.00	51.00	48.00	42.00	39.00	36.00
Sodium bicarbonate	-	-	2.83	5.65	11.30	14.13	16.96
Citric acid anhydrous	-	-	2.17	4.35	8.70	10.87	13.04
MCC PH101	-	-	10.00	10.00	10.00	10.00	10.00
Total	100.00	100.00	100.00	100.00	100.00	100.00	100.00

Remarks: IBU = plain tablet consists of only IBU, IP = 40% by weight of ibuprofen in eutectic tablet, E = % by weight of effervescent agents (the mixture of sodium bicarbonate and citric acid anhydrous in ratio of 1:1.3 by weight) in each formulation such as IP5E stands for formulation containing IP and 5% by weight of effervescent agents.

**Table 2:** Rate of wetting, gel formation, erosion and dissolution parameters of eutectic effervescent tablets in various testing media.

Formulation	Wetting Rate (n = 5) (mm/min)		Gel FormationRate (n = 5) (mm/min)		Erosion Rate (n = 5) (mm/min)	
	Distilled Water	0.1 N HCl Buffer	Distilled Water	0.1 N HCl Buffer	Distilled Water	0.1 N HCl Buffer
IBU	0.201 ± 0.025	0.000 ± 0.000	0.000 ± 0.000	0.000 ± 0.000	0.000 ± 0.000	0.000 ± 0.000
IP	0.515 ± 0.017	0.084 ± 0.005	0.184 ± 0.008	0.171 ± 0.004	0.000 ± 0.000	0.000 ± 0.000
IP5E	0.682 ± 0.122	0.159 ± 0.083	0.053 ± 0.004	0.049 ± 0.004	0.072 ± 0.011	0.058 ± 0.009
IP10E	0.823 ± 0.164	0.236 ± 0.091	0.035 ± 0.002	0.025 ± 0.007	0.153 ± 0.063	0.118 ± 0.044
IP20E	1.407 ± 0.201	0.388 ± 0.143	ND	ND	0.216 ± 0.070	0.194 ± 0.068
IP25E	1.420 ± 0.332	0.485 ± 0.148	ND	ND	0.242 ± 0.069	0.283 ± 0.055
IP30E	1.459 ± 0.311	0.516 ± 0.187	ND	ND	0.301 ± 0.084	0.375 ± 0.092

Remarks: ND = not determined.

**Table 3:** Mathematical modeling of eutectic effervescent tablets in 0.1 N HCl buffer pH 1.2.

Formulation	Kinetic Modeling	Criteria for Model Selection			Kinetic Parameters		
		R^2^	AIC	MSC			
IBU	F_0_ modified Korsmeyer–Peppas	0.9883	−30.5054	4.0201	K_KP_ = 0.1326	n = 0.8168	F_0_ = 0.0000
	Peppas–Sahlin	**0.9926**	**−35.5699**	**4.4805**	**k_1_ = −0.2924**	**k_2_ = 0.3694**	**m = 0.3023**
IP	F_0_ modified Korsmeyer–Peppas	0.9137	54.3542	1.8752	K_KP_ = 7.5186	n = 0.4476	F_0_ = 0.0000
	Peppas–Sahlin	**0.9841**	**35.4159**	**3.5978**	**k_1_ = 5.1761**	**k_2_ = −0.2349**	**m = 0.8300**
IP5E	F_0_ modified Korsmeyer–Peppas	0.8517	62.8388	1.2833	K_KP_ = 10.8410	n = 0.3659	F_0_ = 0.0000
	Peppas–Sahlin	**0.9593**	**48.5215**	**2.5848**	**k_1_ = 8.5832**	**k_2_ = −0.5887**	**m = 0.7207**
IP10E	F_0_ modified Korsmeyer–Peppas	0.8072	64.8453	0.8465	K_KP_ = 16.0896	n = 0.2479	F_0_ = 0.0000
	Peppas–Sahlin	**0.9472**	**50.4698**	**2.1534**	**k_1_ = 15.3681**	**k_2_ = −1.7729**	**m = 0.5647**
IP20E	F_0_ modified Korsmeyer–Peppas	0.8979	55.0850	0.8593	K_KP_ = 23.6146	n = 0.1225	F_0_ = 0.0000
	Peppas–Sahlin	**0.9385**	**49.5094**	**1.3662**	**k_1_ = 29.2022**	**k_2_ = −6.4220**	**m = 0.3129**
IP25E	F_0_ modified Korsmeyer–Peppas	0.8609	61.5988	0.7086	K_KP_ = 25.5091	n = 0.1369	F_0_ = 0.0000
	Peppas–Sahlin	**0.9299**	**53.6240**	**1.4336**	**k_1_ = 30.1941**	**k_2_ = −6.0546**	**m = 0.3633**
IP30E	F_0_ modified Korsmeyer–Peppas	0.9956	20.4575	3.8429	K_KP_ = 26.2569	n = 0.1183	F_0_ = 0.0000
	Peppas–Sahlin	**0.9973**	**16.1655**	**4.2330**	**k_1_ = 31.4441**	**k_2_ = −5.3375**	**m = 0.1751**

Remarks: R^2^ = coefficient of determination; k = rate constant; F_0_ = initial fraction of the drug in the solution resulting from a burst release; AIC = Akaike information criterion; MSC = Model selection criterion.

## Data Availability

The data presented in this study are available on request from the corresponding author.
